# HIV-Specific Antibodies Capable of ADCC Are Common in Breastmilk and Are Associated with Reduced Risk of Transmission in Women with High Viral Loads

**DOI:** 10.1371/journal.ppat.1002739

**Published:** 2012-06-14

**Authors:** Jennifer Mabuka, Ruth Nduati, Katherine Odem-Davis, Dylan Peterson, Julie Overbaugh

**Affiliations:** 1 Division of Human Biology, Fred Hutchinson Cancer Research Center, Seattle, Washington, United States of America; 2 Program of Pathobiology, Department of Global Health, University of Washington, Seattle, Washington, United States of America; 3 Department of Pediatrics, University of Nairobi, Nairobi, Kenya; 4 Public Health Sciences, Fred Hutchinson Cancer Research Center, Seattle, Washington, United States of America; Harvard University, United States of America

## Abstract

There are limited data describing the functional characteristics of HIV-1 specific antibodies in breast milk (BM) and their role in breastfeeding transmission. The ability of BM antibodies to bind HIV-1 envelope, neutralize heterologous and autologous viruses and direct antibody-dependent cell cytotoxicity (ADCC) were analyzed in BM and plasma obtained soon after delivery from 10 non-transmitting and 9 transmitting women with high systemic viral loads and plasma neutralizing antibodies (NAbs). Because subtype A is the dominant subtype in this cohort, a subtype A envelope variant that was sensitive to plasma NAbs was used to assess the different antibody activities. We found that NAbs against the subtype A heterologous virus and/or the woman's autologous viruses were rare in IgG and IgA purified from breast milk supernatant (BMS) – only 4 of 19 women had any detectable NAb activity against either virus. Detected NAbs were of low potency (median IC50 value of 10 versus 647 for the corresponding plasma) and were not associated with infant infection (p = 0.58). The low NAb activity in BMS versus plasma was reflected in binding antibody levels: HIV-1 envelope specific IgG titers were 2.2 log_10_ lower (compared to 0.59 log_10_ lower for IgA) in BMS versus plasma. In contrast, antibodies capable of ADCC were common and could be detected in the BMS from all 19 women. BMS envelope-specific IgG titers were associated with both detection of IgG NAbs (p = 0.0001)and BMS ADCC activity (p = 0.014). Importantly, BMS ADCC capacity was inversely associated with infant infection risk (p = 0.039). Our findings indicate that BMS has low levels of envelope specific IgG and IgA with limited neutralizing activity. However, this small study of women with high plasma viral loads suggests that breastmilk ADCC activity is a correlate of transmission that may impact infant infection risk.

## Author Summary

In the absence of intervention, only about one third of infants born to HIV-1 infected mothers who are continuously exposed to maternal breast milk over prolonged periods get infected. This observation raises the possibility that immune factors in infected women play a role in limiting HIV-1 transmission. Identifying factors associated with reduced HIV-1 transmission risk will improve our understanding on the potential correlates of protection that should be the focus of generating effective immunogens and vaccination protocols. Here we assessed the functional role of breast milk antibodies in a group of women with high plasma viral loads and systemic NAbs and determined that overall, breast milk contains low levels of neutralizing antibodies when compared to plasma. In contrast, we observed a robust non-neutralizing activity in breast milk that was associated with infant infection status. Our study adds to the growing evidence of a potential role of non-neutralizing antibodies in limiting HIV-1 transmission and calls for more attention to this arm of the HIV-1 response.

## Introduction

Breast milk (BM) can be a vehicle for transmission of various pathogens, but the risk of infant infection is balanced by the potential clinical benefit of BM, which provides significant passive immunity and protection against many infectious agents [1]–[4]. In the case of HIV-1, exposure to virus through breastfeeding accounts for almost half of the 30–40% of vertical transmissions that occur in untreated, breastfed infants of HIV-1 positive women [5]–[7]. Replacement feeding, avoidance of breastfeeding and reduced BM exposure by early weaning can significantly reduce BM transmission, however, these interventions have been associated with significant increase in infant morbidity and mortality [8]–[13]. Additionally, HIV-1 infected as well as exposed uninfected infants who do not breast feed have been shown to exhibit stunted growth [14], [15]. These observations highlight the challenges facing HIV-1 infected women in sub- Saharan Africa where prolonged breastfeeding could lead to HIV-1 transmission but no breast feeding could increase the risk of morbidity and mortality resulting in a diluted benefit of HIV-1 free survival [16]–[18]. Consequently, greater understanding of BM protective factors in HIV-1 infection may open promising new ways to make breastfeeding safe for infants born toHIV-1 infected women.

Approximately 15–20% of infants born to all HIV-1+ mothers in chronic infection acquireHIV-1 through BM [6], [7], [19], [20]. This relatively low infection rate despite continued exposure suggests that either BM infectivity is low or that antiviral factors in BM may play a role in modulating transmission and/or acquisition of HIV-1 via the oral mucosa. Indeed, antiviral innate immune factors present in BM such as alpha defensins, bile salt-stimulated lipase, lactoferrin, and mucins have all been associated with modulating the risk of BM transmission [21]–[23]. BM is also composed of both innate and activated adaptive immune cells, presumably derived from other mucosal sites such as the gut associated lymphoid tissue. Indeed, HIV-1 specific CD8 T cells and B cells have been reported in BM [24]–[26], but to date there have been no published studies that have explored the association between the functional immune responses in BM and risk ofHIV-1 transmission through breastfeeding.

Vertical transmission, including BM transmission, is characterized by a transmission bottleneck [27]–[39]. In mother- to-child transmission (MTCT), it has been suggested that this bottleneck is in part a result of selection pressure from Nabs because the viruses that are transmitted tend to be relatively insensitive to neutralization by maternal autologous antibodies (Abs), even in mothers who harbor viruses with a range of neutralization sensitivities[32], [39]. Consistent with the hypothesis that adaptive immunity plays a role in MTCT, several studies comparing levels of maternal plasma neutralizing antibody (NAb) titers reported that transmitting (T) mothers have lower levels of NAb in plasma compared to non-transmitting (NT) mothers [27], [32], [36]–[42] suggesting that maternal NAb may contribute to protection of the infant. However, the results of these studies are not consistent, particularly with respect to a role for NAb in protection by different routes of transmission [43]–[45]. Moreover, a recent study of passive Absin 100 HIV-1 exposed infants did not find evidence for a protective effect of broadly NAb on infant infection [46].

Until recently, most studies of BM HIV-1 Abs focused primarily on determining the association between the levels or presence of binding Abs to envelope (env) proteins and transmission. Several studies that have focused on BM IgG and IgA have showed no association between levels of these antibodies and transmission [47], [48]. Notably, infant infection status in these early studies was determined by serology and/or clinical manifestation of AIDS, a situation that could result in misclassification of infant infection status. A more recent study that determined infant infection by DNA PCR showed increased levels of BM IgA in T compared to NT women suggesting that, rather than providing protection, BM HIV-1 env specific soluable IgA, is associated with increased risk of transmission [49]. However, all these studies used subtype B env proteins, in some cases from lab adapted viruses to detect HIV-1 binding Abs despite being conducted in sub-Saharan Africa where such variants are not typical of transmitted strains of HIV-1 [49], [50]. Taken together, the results from BM binding studies have not provided clear evidence of a role of BM Abs in vertical transmission.

BM Abs could provide benefit by directly neutralizing the virus within the milk or by non-neutralizing mechanisms such as antibody dependent cellular cytotoxicity (ADCC)that target infected cells. This could result in reduced levels of infectious cell-free virus and BM infected cells, which are both correlates of BM transmission [51]–[54]. The potential of Abs in BMto neutralize HIV-1 and/or mediate ADCC has only very recently been examined, and in this study of ARV-exposed, subtype C-infected women in Malawi, NAbs were detected in about half of the BM samples while ADCC activity was present in all BM samples obtained at 1 month after delivery [55]. There have been no studies to-date looking at BMS samples obtained from untreated T and NT women, particularly in colostrum and early milk, which is relevant given that virus levels are highest in colostrum [51]and the majority of BM transmissions occur early in life [6], [20]. There has also been no study looking at how these BM Abs function in relation to MTCT.

We evaluated neutralizing, binding and ADCC activity in BMS or BMS-derived IgG and IgA and matched plasma from antiretroviral (ARV) naïve T and NT mothers with high plasma viral loads and systemic NAbs. Our data shows that BM Nabs are rare and their levels are significantly lower than in plasma. However, we report a high frequency of ADCC activity in BMS that was significantly higher in NT women compared to T women. These data suggest that BMADCC mediating Abs but not Nabs may play a role in modulating HIV-1 transmission.

## Materials and Methods

### Study subjects and sample collection

Women enrolled in a randomized clinical trial comparing breastfeeding to formula feeding in Nairobi Kenya provided BMS samples used in this study [6]. Subjects received coded identification numbers at the clinic and therefore BMS samples were anonymous to laboratory personnel. The ethical review committees of the University of Nairobi, the University of Washington and the Fred Hutchinson Cancer Research Center approved this study and the Kenyan ministry of health gave permission for the original study to be conducted. The methods for enrollment, counseling and follow up have been described elsewhere [6], [51]. Briefly, HIV-1 positive women were enrolled at 32 weeks gestation and blood samples were taken for viral load and CD4 count testing. Maternal blood, breast milk samples, and infant blood samples were collected within the first week post-delivery, at 6 weeks, 14 weeks, 6 months and quarterly thereafter until 2 years. Infant HIV-1 status was determined using DNA PCR [56]. Breast milk samples were centrifuged to remove the lipid layer and the supernatant was stored at −70°C before being shipped either on dry ice or in liquid nitrogen to Seattle, Washington for long term storage at −70°C until use. Plasma and BM viral loads were determined using the Gen-Probe HIV-1 RNA assay (Gen-Probe, La Jolla, Calf) [51], [57]. Breastmilk samples used in this study were chosen as the first available breastmilk sample after delivery for each woman and the reported breastmilk viral loads are contemporaneous.

### Breast milk IgG Ab purification

BMS IgG was purified using NAb Protein G spin columns (Pierce, Biotech, Rockford, IL), with minimal changes to the manufactures instructions. Briefly, 250 ul of heat-inactivated BMS was added to 250 ul of binding buffer and the mixture was added to a protein G column followed by incubation at room temperature (RT) with end over end mixing for 30 min. Thereafter, the column was centrifuged to obtain the IgG flow through (IgG stepFT) which was saved for subsequent IgA purification. The column with bound Ab was washed 3 times with 400 ul of binding buffer. Bound Ab was eluted with 1 ml of elution buffer (pH 2.8) and the eluate was neutralized by adding 100 ul of 1 M Tris. HCl (pH 8.5). Thus, the final purified IgG Ab was diluted 4-fold relative to the original BMS. The final eluted IgG and IgA was retained at a 1∶4 dilution of the original BMS and this was used undiluted in further neutralization assays. Coomassie blue staining (Simply Blue, Invitrogen) and ELISAs using Human IgG ELISA kit (E-80G) and human IgA ELISA kit (E-80A) (Immunology Consultants laboratory, Newberg, OR) were used to confirm the purity of Ab fractions.

### Breast milk IgA Ab purification

BMS IgA was purified from the IgG step FT using the method outlined by Hirbod et.al with some modifications [58]. Spin columns (Thermo) were packed with 400 ul of immobilized jacalin (Pierce biotech, Rockford, IL) and washed 3 times with 400 ul of PBS to equilibrate. The column was then loaded with 500 ul of the IgG step FT and incubated on an end over end roller for 2 hours at RT. After incubation, the column was centrifuged and a final flow through (FT- fraction lacking IgG and IgA) was collected and stored for analysis. The column was washed 3 times with PBS followed by a 3-hour incubation with 500 ul of 1 M Melibiose to elute bound IgA. The column was further washed with another 500 ul of elution buffer to maximize recovery and bring the final dilution of purified IgA fraction to 1∶4 relative to the original BMS, similar to the IgG fraction. As before, coomassie staining and ELISA were used to confirm the purity of Ab fractions.

### Generation of HIV-1 Env genes and corresponding pseudoviruses

The subtype A HIV-1 envQ461.d1 was cloned directly from peripheral blood mononuclear cells (PBMCs) of a recently infected Kenyan woman as described previously [59]. Autologous PBMC and BM cell derived clones have either been previously described or were obtained using the same protocol [39], in some cases with modification of primers to allow amplification of the HIV-1 variant in that particular sample (primers are available upon request). Plasmid DNA encoding the env of interest and a plasmid encoding an env-deficient HIV-1 subtype A proviral DNA, Q23Δenv [60], were co-transfected into 293T cells at a 1∶2 molar ratio to generate pseudotyped viral particles as described [39], [61]. Virus was harvested 48 hrs post-transfection and the infectivity was determined by single round infection of TZM-bl cells as described [39]. Pseudoviruses were also generated using Q23Δenv and simian immunodeficiency virus clone 8 (SIV) [62]oramphotropic murine leukimia virus (MuLV)envelope clones [63].

### Neutralization assays

Neutralization was assessed by determining infection of a reporter cell line, TZM-bl, as previously described [39]. Briefly, 500 infectious particles were incubated with 2-fold serial dilutions of heat inactivated plasma or BMS, purified BMSIgG or IgA fraction, FT fraction or media only in a total volume of 50 ul at 37°C for 1 hour. TZM-bl cells in 100 ul of growth medium containing 30 ug/ml of diethylaminoethyl-dextran were then added. After 48 hours, neutralization was determined by measuring β-galactosidase activity present in the TZM-bl cell lysate. For each virus/Ab combination, at least two independent experiments were performed. Each experiment was performed intriplicate for plasma and BMS or duplicate for purified BMSAb fractions. Median inhibitory concentrations (IC50s) were defined as the reciprocal dilution of plasma, BMS or purified antibody that resulted in 50% inhibition, calculated by interpolation of the linear portion of the neutralization curve on the log_2_ scale as previously described [39], [61]. Plasma and BMS samples were tested at 1∶100 and 1∶20 dilution respectively, while purified BMSAb fractions were tested at 1∶8 dilution (a 2-fold dilution of the recovered purified fractions that were diluted 4 fold during processing). For the purposes of analysis, in cases in which the IC50s were less than the lowest dilutions tested, the midpoint value between the lowest dilution and zero was assigned. IC50s from replicate experiments were averaged by the geometric mean. Here IC50s indicate the geometric mean IC50 estimates [64].

### ELISAs for total and HIV-1 Env specific IgG and IgA

Human IgG ELISA kit (E-80G) and human IgA ELISA kits (E-80A) (Immunology Consultants laboratory, Newberg, OR) were used to determine the levels of total IgG and IgA in un-purified BMS and plasma samples according to the manufacturer's instructions.

HIV-1env specific ELISAs were performed using the protocol outlined by Sather et.al with minimal modifications [65]. Briefly, Immulon 2HB ELISA plates were coated with 25 ng/well of a HIV-1 subtype A Q461.d1 soluble trimeric gp140 protein purified as described in [66] in 0.1 M NaHCO_3_, pH 9.4 overnight at room temperature. Plates were blocked in phosphate buffered saline (PBS), supplemented with 10% dry milk and 0.3% Tween-20 for 1 hr at 37°C. Unpurified BMS and plasma samples were diluted in 10% dry milk, 0.03% Tween in PBS. For detection of HIV-1env specific IgG and IgA, BMS samples were diluted at 1∶100 and were titrated 2-fold up to a maximum dilution of 12,800. In cases where an end point titer could not be determined at this dilution, samples were diluted further up to a final dilution of 104,200. For HIV-1 env specific plasma IgG, samples were diluted at 1∶100,000 followed by a 2-fold titration up to a maximum dilution of 12,800,000 while for IgA samples were initially diluted 1∶200 followed by a 2-fold dilution up to 25,600. Samples were loaded in duplicate wells and incubated for 1 hr at 37°C. Plates were washed in a plate washer and bound IgG Ab was detected at 37°C for 1 hr with goat anti-human IgG- horseradish peroxidase (HRP) (Bio-Rad, Hercules, CA) diluted 1∶3000 while IgA was detected by goat anti human IgA HRP(Invivogen, San Diego, CA) diluted 1∶4000. Plates were developed with 50 ul of 1-Step Ultra TMB-ELISA solution (Pierce Biotech, Rockford. IL) and stopped with 50 ul 1 N H_2_SO_4_. Absorption at 450 nm was read on an EL808 Ultra Microplate Reader (Bio-TEK Instruments.inc). In this study, end point titer (EPT) was defined as the BMS or plasma reciprocal dilution at which the average OD value was greater than or equal to two times the average OD value of background.

### Rapid fluorescence-antibody dependent cellular cytotoxicity assay (RF-ADCC)

The ability of BMS and their matched plasma to mediate ADCC activity was determined as described by Gomez-Roman et.al with a few modifications [67]. Briefly, CEM. NKr cells, a natural killer resistant cell line (AIDS Research and Reference Reagent Program, NIAID,NIH) were double stained with a membrane dye, PKH-26(Sigma, St. Louis, MO, USA) and a viability dye, carboxyfluorescein diacetate, succinimidyl ester (CFSE) (Molecular Probes, Eugene, OR, USA) as recommended by the manufactures. After staining, 1×10^5^ cells were coated for 1 hr at RT with 1.5 ug HIV subtype Agp120 protein obtained from an infant in the Nairobicohortat 6 weeks post-infection (BL035) [39]. Coated cells were then washed once and resuspended in 1 ml of RPMI with 10%FBS. Five thousand coated or uncoated CEM. NKr cells were added to the appropriate duplicate wells containing 100 ul of 1∶100 or 1∶1000 heat inactivated BMS or plasma respectively. Similar experiments were performed using media only or HIV IgG (NIH AIDS Research, Germantown, MD, USA)as negative and positive controls, respectively. The antibody-target cell mixture was incubated at RT for 10 min to allow the antibody to interact with the antigen on the surface of target cells. Following incubation, 50 ul of effector cells (HIV negative donor PBMCs) were added to the mixture at an effector to target cell (*E/T*) ratio of 50∶1 and incubated for 4 hours at 37°C. For all 19 BMS and plasma samples, PBMCs from the same donor were used in parallel assays. Cells were then washed and fixed in 150 ul of 1% paraformaldehyde-PBS and stored at 4°C overnight. Fixed cells were analyzed within 24 hours of the ADCC assay using a BD LSRII instrument (Becton Dickinson, San Jose, CA, USA). Flow cytometry data was analyzed using Flojo version 9.4.6(Tree Star Inc, Ashland, OR, USA). ADCC percent killing was defined as the percentage of membrane labeled cells (PKH-26^+^) that had lost their viability dye (CFSE^−^) after subtracting two times the level of killing in the media only wells (background), as described in (67).

### Statistical analysis

Odds ratios (OR) for assessment of associations between detection of HIV-1 specific and non-specific activity in BMS and transmission were estimated by Fisher's Exact Test. IC50sfor HIV positive and HIV negative controls were compared by one-sided t-test on the log_2_ scale. All comparisons of Ab total concentrations and HIV-1 env specific titers were based on paired t-tests on the log_10_ scale, noting that differences on the log scale were approximately normally distributed, and corresponding multivariate adjustments were by linear regression. HIV-1 specific titers among those with detected virus neutralization by BMS IgG and IgA were each compared to titers among those with undetected neutralization using Welch's t-test on the log_10_ scale. All correlations were measured by Pearson's product moment correlation coefficient (PPMCC), denoted r, with p-values based on the Student's t approximation for the distribution of the corresponding standardized test statistic. The relationship between maternal clinical correlates and BMS Ab neutralization, HIV-env specific binding titers and ADCC activity were each individually assessed by Welch's t-test with corresponding adjusted estimates by linear regression. Statistical analysis was performed using R 2.13 ISBN 3-900051-07-0 and STATA version 11 edition, (College Station, TX).

## Results

### Characteristics of women in the study

The goal of this study was to determine the presence and functional capacity of BM HIV-specific antibodies and to determine if they impact MTCT. Therefore, we selected women who had high plasma viral loads (greater than the cohort median of 4.6 log_10_)and thus were at increased risk of transmission. Among these women, we identified those who exhibited potent plasma NAb responses (Majiwa and Overbaugh, unpublished data) to maximize the chances of detecting BM NAbs. From this subset of women, we selected those that breast-fed for greater than 3 months to capture cases of BM HIV exposure to the infant. Women whose infants were HIV-1 positive before 6 weeks of life were excluded to ensure that transmission was as a result of BM and not late in-utero, or intra-partum exposure. An additional criteria was that women had available BMS samples collected at less than14 weeks after delivery because this early period is the window within which the majority of BM transmissions occur [6] and protein concentrations are highest [68], [69]. Nineteen women with a median CD4 count of 360 cells/uL met these criteria. The median plasma and BM viral loads were5.22 and 2.44 log_10_ respectively, an ∼2-log difference that was also observed in the larger cohort [51]. Nine of these women transmitted HIV-1 to their infants via BM at various time-points postpartum (Table 1).

**Table 1:** The characteristics of transmitting and non-transmitting women in the study and the neutralization IC50s of their plasma and BMS.

							IC50s				
							Plasma^a^		BMS^b^		
ID Number	Viral Subtype	CD4 Count	Log_10_ Plasma VL^c^	Log_10_ BMS VL^c^	Infant-Infection Wk^d^	Visit Wk^e^	HIV^f^	SIV	HIV	SIV	MLV
Transmitting MB885	A	136	4.78	1.93	6	0	535	50	85	83	95
Women MC046	A	255	5.05	3.17	6	0	1084	50	23	30	27
MF520	A	511	5.59	2.73	15	1	327	50	23	25	26
MF535	D	690	5.53	2.37	6	14	3144	50	21	10	10
MI206	A	262	5.12	2.27	6	0	751	50	22	10	24
MJ412	C	293	4.86	2.9	6	0	283	50	10	29	33
MJ613	A	104	5.64	2.96	6	1	751	50	10	10	24
MJ776	A	385	5.44	4.24	6	0	510	50	28	10	30
MM596	nd^g^	392	5.75	2.26	6	6	469	50	10	10	10
Non-Transmitting MA411	A	416	5.5	2.76	na^h^	0	1200	50	38	33	38
Women MB727	C	416	4.70	2.54	na	8	314	50	10	10	10
MB807	A	217	4.78	3.62	na	0	989	50	10	10	10
MG540	A	285	5.6	3.95	na	0	762	50	37	27	22
MH230	A	651	5.02	2.79	na	14	647	50	22	10	10
MK371	D	352	4.62	bd^i^	na	2	354	50	10	10	10
ML055	D	213	5.18	2.76	na	0	1107	50	10	10	45
ML267	nd	551	5.68	3.77	na	0	185	50	10	10	10
MM471	A	360	5.26	3.36	na	8	1963	50	10	10	10
MP199	A	389	5.22	2.73	na	6	1253	50	10	10	28

a Plasma neutralization assays were performed at a starting dilution of 1∶100; an IC50 of 50 was assigned in cases where 50% neutralization was not achieved.

b BMS neutralization assays were performed at a starting dilution of 1∶20; an IC50 of 10 was assigned in cases where 50% neutralization was not achieved.

c Viral Load.

d Indicates week since delivery when infant was first HIV-1 DNA positive.

e Indicates time-point after delivery at which BM sample was obtained.

f Q461.d1.

g Not done.

h Not applicable.

i Below detection.

### Non-specific inhibition of viruses by BMS

**Figure 1:**
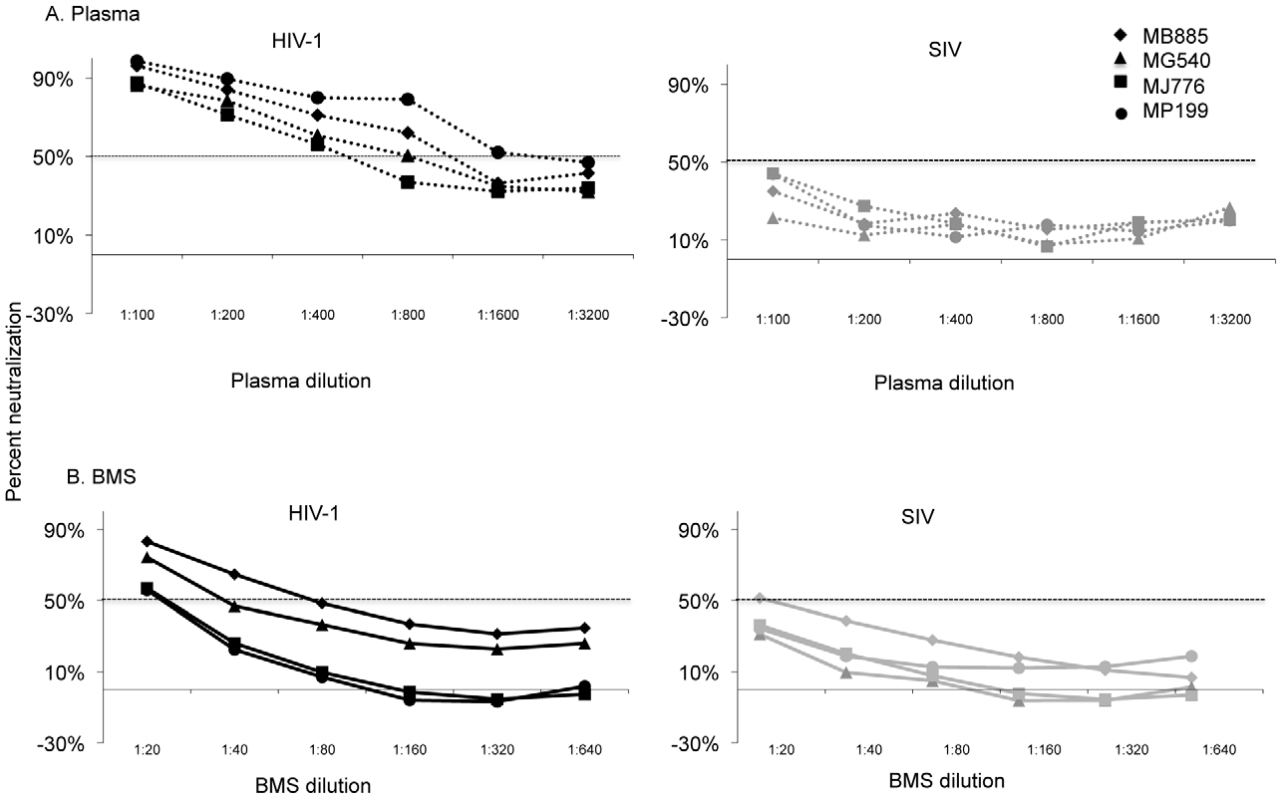
Neutralization potency of plasma and BMS from four mothers against heterologous virus. The graphs show percent neutralization versus plasma (A) or BMS (B) dilution. Results using pseudovirus generated with heterologous Q461.d1 env (HIV-1 in black lines) are shown in the left graph and with SIVMneCl8 (SIV in grey lines) are shown in the right graph. The corresponding symbol for the data from each of the four mothers is shown in the upper right corner. The 50% neutralization level is shown with a dotted line. The results are from triplicate testing and are representative of at least two independent experiments. The average IC50s for the two experiments for all 19 women is reported in Table 1.

The ability of heat inactivated BMS to neutralize virus bearing a highly sensitive env variant isolated from a Kenyan woman soon after her infection was determined. This heterologous HIV-1 subtype A env variant, Q461.d1, was chosen because >90% of plasma from individuals in the region showed detectable neutralization of this virus at a 1∶100 plasma dilution [70]. The results with plasma from 4 representative women are shown in Figure 1A. All4 plasma samples neutralized Q461.d1 with IC50 values of ∼500 or greater. Importantly, 50% inhibitory activity was not achieved when testing plasma samples against SIV suggesting that the neutralization response was specific to HIV-1. Overall, virtually all19 plasmas displayed potent HIV-1 specific neutralization, with IC50s ranging from 185 to 3144 (Table 1).

We could not detect HIV-1 neutralization in any of the BMS at a similar starting dilution as plasma (1∶100 data not shown). At a very low starting dilution (1∶4) there was substantial non-specific inhibition of SIV and MuLV and preliminary assays suggested potential cytotoxic effect of more concentrated BMS, as reported previously [71]. BMS was therefore tested at a starting dilution of 1∶20, hence 5× more concentrated compared to plasma. Results from BMS of 4 representative women against Q461.d1 and SIV are shown in Figure 1B. While a low level of inhibition of HIV-1 was observed with some BMS such as MJ776 and MP199, there was little difference in the magnitude of BMS neutralization of Q461.d1 and SIV in all 4 cases. Among all 19 women, 9 BMSs - 6 from T and 3 from NT women - showed HIV-1 inhibition with IC50 values ranging from 21–85; there was no detectable inhibition by BMS from 3 T and 7 NT women. BMS from the majority of women also inhibited SIV and MuLV pseudoviruses, with IC50 values ranging from 20–95 (Table 1). A paired comparison of BMS HIV-1 IC50s with the geometric mean of IC50s for corresponding negative control viruses (SIV and MuLV)showed that HIV-1 IC50s were not statistically greater than those of the negative controls (p = 0.44). This observation suggested that the majority of inhibition we observed with BMS was likely not due to HIV-1 specific Abs.

The presence of a non-specific inhibitor of HIV-1 in BMS could nonetheless be relevant to transmission risk. We thus examined the association between detection of non-specific activity and transmission and found that this relationship was not statistically significant (OR = 4.77; 95% CI: 0.51, 71.53; p = 0.17).

### Limited ability of purified BMS IgG and IgA Abs to neutralize heterologous virus

**Figure 2:**
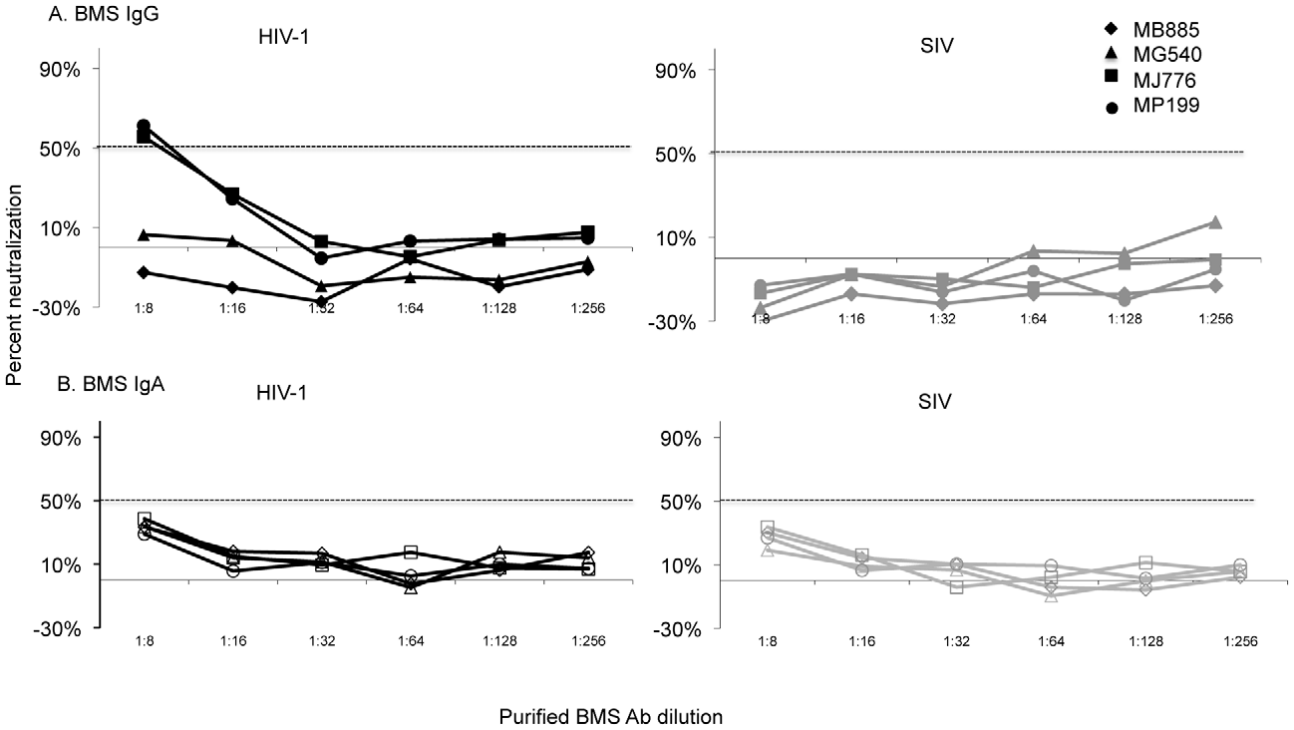
Neutralization potency of purified IgG and IgA from four mothers against heterologous virus. The graphs show percent neutralization versus BMS purified IgG (A) or IgA (B) dilution. The corresponding symbol for the data from each of the four mothers is shown in the upper right corner. Neutralization by IgG and IgA is represented by filled and open symbols, respectively. Results using pseudovirus generated with Q461.d1 env (HIV-1 in black lines) are shown in the left graph and with SIVMneCl8 (SIV in grey lines) are shown in the right graph. The 50% neutralization level is shown with a dotted line. The results are from duplicate testing and are representative of at least two independent experiments. The average IC50s for the two experiments for all 19 women is reported in Table S1.

To determine what portion of the non-specific inhibition observed with unfractionated BMS was due to Abs versus other factors, we separately purified IgG and IgA Abs from BMS for use in the neutralization assays. Bands of the expected sizes for IgG and IgA were observed in the respective purified fractions by coomassie staining and cross contamination between Ab isotype fractions by total Ig ELISA was below detection (data not shown). Purified Ab fractions were tested at a starting dilution of 1∶8, which translated to a dilution 2.5 times higher than the most concentrated BMS we tested (1∶20 dilution). Using the purified BMS IgG fractions, neutralization of greater than 50%was detected in only 2 (subjects MJ776 and MP199) of 19 purified BMS IgG tested, with IC50s of 9.4 and 9.9 respectively. (These two examples are shown in figure 2A and a summary of the 19 in Table S1). Of these women MJ776 transmitted HIV-1 to the infant while MP199 did not. In contrast, there was no detection of neutralization by purified BMS IgA fractions tested (Results from 4 representative women are shown in figure 2B and a summary of the 19 in Table S1). Importantly, purified BMS IgG and IgA fractions did not inhibit viruses pseudotyped with SIV env including the two BMS IgG fractions from subjects MJ776 and MP199, which had detectable neutralization of virus pseudotyped with Q461.d1env (Figure 2A, B, and Table S1). The FT fraction, which contained undetectable levels of BM IgG and IgA both by ELISA and coomassie staining, (data not shown) retained the non-specific activity displayed by BMS (Table S1).

### Limited ability of purified BMS IgG and IgA Abs to neutralize autologous blood and breast milk-derived virus

**Figure 3:**
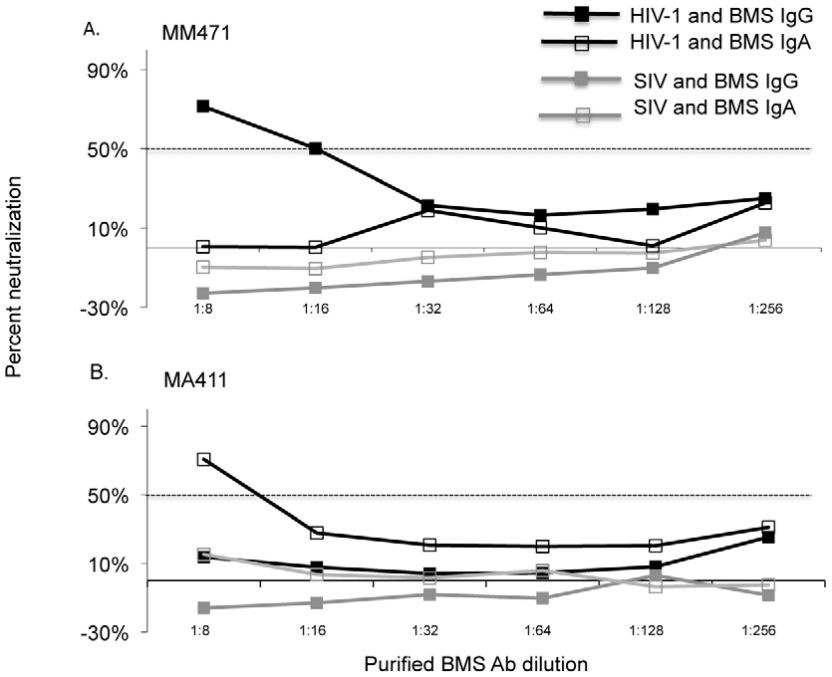
Neutralization potency of IgG and IgA from two mothers against autologous virus. Representative graphs showing percent neutralization versus BMS purified IgG or IgA dilution. (a) Neutralization by IgG and IgA fractions from subject MM471 and (b) neutralization by IgG and IgA fractions from subject MA411. IgG (filled square) and IgA (open square) responses against pseudovirus generated with autologous HIV-1 env are shown in black lines and against SIVMneCl8 (SIV) are shown in grey lines. The 50% neutralization level is shown with a dotted line. The results are from duplicate testing and are representative of at least two independent experiments.

To ensure that we were not missing NAb responses by using a heterologous virus, we examined the ability of BMSAb fractions to neutralize autologous virus in a subset of the 19 women. BMS IgG and IgA Ab fractions and FT from a total of 8 women were each tested against 2 pseudoviruses bearing autologous env variants from blood [39]. Of the 8 women, 2 women both NTs, showed low potency neutralization of the blood-derived autologous virus to one of the two viruses tested. MM471 displayed low neutralization potency with anIC50 of 15against one of her autologous viruses when using IgG but not the IgA fraction (representative experiment is shown in figure 3A). In contrast, MA411 displayed low neutralization potency with an IC50 of 9 against one of the autologous virus with IgA but not with IgG fractions (a representative experiment is shown in figure 3B). BMS IgG and IgA fractions from the remaining six women, all Ts did not neutralize their respective autologous viruses above 50%. Autologous viruses for MJ776 and MP199 were not available for testing

The ability of plasma and BMS purified Ab to neutralize variants obtained from BM was also determined for two subjects MF535 (T) and ML055 (NT). Autologous plasma from MF535 and ML055 diluted at 1∶100 neutralized the respective BM viruses withIC50s of 152 and 718, respectively. In contrast, there was no detectable neutralization by BMAb fractions against these autologous BM viruses (data not shown).

### BMS IgG total and HIV-1 Env specific titers are lower than plasma IgG

**Figure 4:**
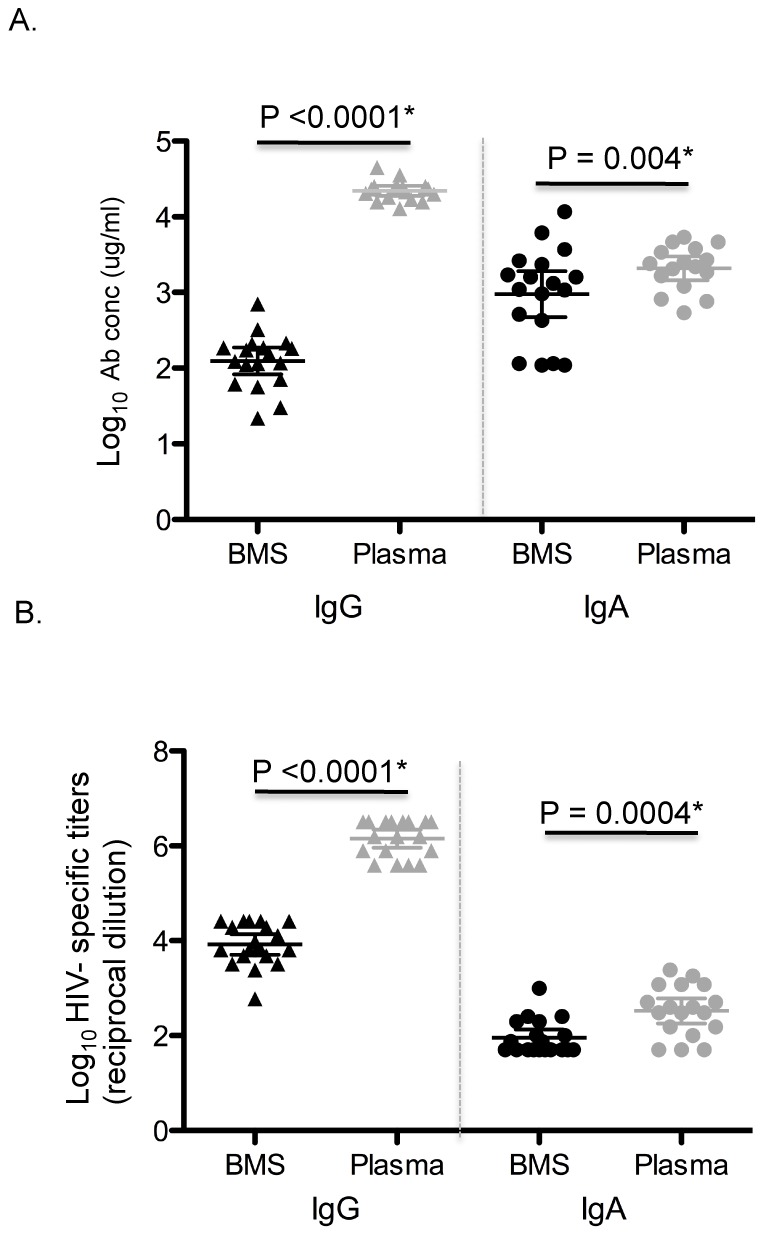
Levels of total and HIV-1 env specific IgG and IgA in unfractionated BMS and plasma. (A)Total IgG and IgA in unfractionated BMS and plasma. The Y-axis shows the log_10_ Ab conc (ug/ml) and the X-axis shows the sample type and Ab isotype. Black and grey symbols denote BMS and plasma, respectively. Triangles and circles represent IgG and IgA, respectively. (B) Unfractionated BMS and plasma HIV-1 env specific IgG and IgA titers. The Y-axis shows the log_10_ HIV-1 env specific titers (reciprocal dilution) and the X-axis shows the sample type and Ab isotype. Symbols are as described for A.

To determine if low NAbs in BMS reflected lower total BM Ab levels, we measured the levels of total and HIV-1envspecific IgG and IgA Abs in BMS and compared them to plasma (Figure 4). The levels of total BMS IgG were 0.88 log_10_ lower than BMS IgA(p<0.0001) (Figure 4A, black symbols). This is in contrast to plasma, where the IgG levels were found to be 1.02 log_10_ higher than IgA (p<0.0001) (Figure 4A, grey symbols). There was a pronounced difference between the magnitude of total IgG in BMS and plasma with BMS total IgG being2.25 log_10_ lower than plasma IgG (p<0.0001). In contrast, the total IgA levels in plasma were only slightly higher than in BMS, with a modest 0.39log_10_ difference between BMS and plasma (p = 0.004). We found statistically significant correlation between total BMS IgG and plasma IgG (r = 0.67; p = 0.0034)while the levels of BMS total IgA correlated with total plasma IgA (r = 0.78; p = 0.0003). There was no significant correlation between BMS total IgG and BMS total IgA (r = 0.39; p = 0.10) (Table S2).

Next, we determined HIV-1 env specific IgG and IgA titers in unfractionated BMS and plasma against soluble gp140 protein derived from the subtype A variant, Q461.d1, that was used for the neutralization studies (Figure 4B). HIV-1 env specific IgG titers were obtained in 100% of BMS and plasma samples. In contrast, HIV-1 env specific IgA titers were obtained in 50% of BMS and 90% of plasma samples; the rest were below the cut off value for EPT as defined in this study. BMS HIV-1 env specific IgG titers were 1.96 log_10_ higher compared to env specific IgA (p<0.0001) (Figure 4B, black symbols). Similarly, HIV-1 env specific IgG titers in plasma were higher by 3.63 log_10_ when compared to the env specific IgA titers (p<0.0001) (Figure 4B, grey symbols). Overall, similar to what we found for total IgG levels, BMS HIV-1 env specific responses were 2.22 log_10_ lower compared to that in plasma (p<0.0001) (Figure 4B). For HIV-1 env specific IgA, the log_10_ difference between BMS and plasma was 0.59 (p = 0.0004) (Figure 4B). BMS HIV-1 env-specific IgG titers were correlated with plasma HIV-1 env specific IgG titers (r = 0.81; p<0.0001)and BMS total IgG (r = 0.76; p = 0.0003). There was no statistically significant correlation between BMS HIV-1 env specific IgG titers and BMS HIV-1 env specific IgA (Table S2). Similar to BMS HIV-1 env specific IgG titers and BMS total IgG, BMS HIV-1 env specific IgA titers and BMS total IgA levels were also positively correlated (r = 0.69; p = 0.015) (Table S2.)

We examined the relationship between the levels of HIV-1 env specific titers in BMS and detection of neutralizing activity. The three women with IgG neutralizing activity had a log_10_ IgG titer of 4.41 as compared to a mean of 3.83 among non-IgG-neutralizers (p = 0.0001). The one woman with IgA NAbs also had the highest IgA env specific titer, which was1.10log_10_ greater than the group median. (Figure. S1).

### ADCC activity is common in BMS and it correlates with HIV-1 env specific IgG titers

**Figure 5:**
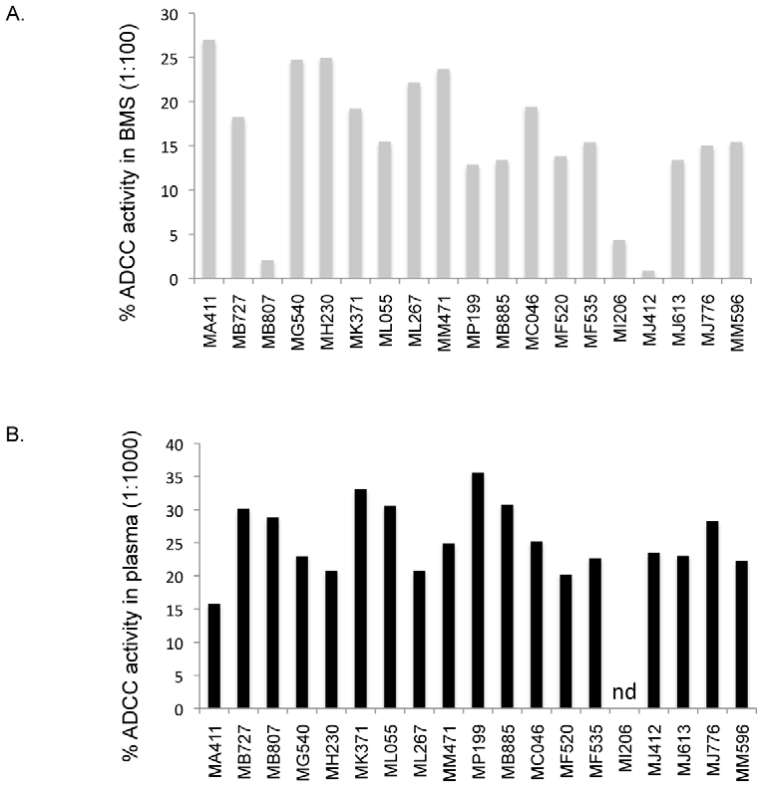
ADCC activity in BMS and plasma. Percent ADCC activity in BMS(A) and plasma (B). BMS was tested at a 1∶100 dilution and plasma at a 1∶1000 dilution. The subject ID for the corresponding ADCC measure is shown below each bar. The results are from duplicate testing and are an average of at least two independent experiments each done using effector cells from a single donor. nd indicates not done.

**Figure 6:**
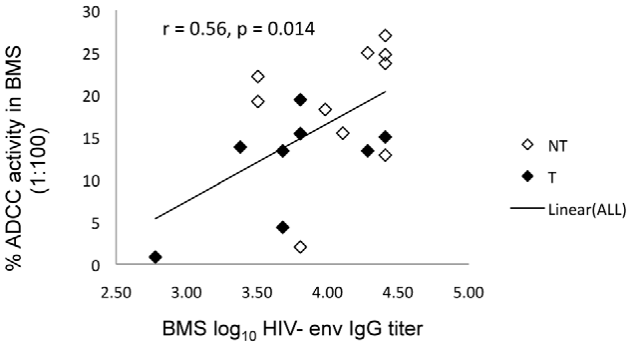
ADCC activity in relation to HIV-1 Env specific IgG titers in BMS. The Y-axis shows % ADCC activity in BMS (1∶100) and the X-axis shows the log_10_ BM HIV-1 env specific IgG titers. Filled and open symbols represent transmitting and non-transmitting women, respectively. The trend line ‘Linear (ALL)’ is the regression line including both transmitting and non-transmitting women.

We determined the capacity of BMS binding antibodies and their matched plasma to mediate ADCC. The appropriate BMS and plasma dilution for the ADCC assay was determined by testing serial 10-fold dilutions of 4 representative BMS and plasma in the ADCC assay. The dilution that permitted detection of HIV-specific ADCC activity above background levels, but did not yield inhibition of ADCC activity that can occur with more concentrated samples [72] was chosen for testing (1∶100 for BMS and 1∶1000 for plasma). Using a single dilution also allowed us to test all 19 BMS and plasma samples with effector cells obtained from a single PBMC donor, which is critical for avoiding bias due to differences in effector cell activity observed from donor to donor. Overall, ADCC activity was detected in all BMS and plasma samples tested (Figures 5 A and B). BMS ADCC mediated killing ranged from 1–27% (median,15%) while that of plasma ranged from 16–36% (median, 24%). BMS ADCC activity was correlated with gp140 env specific IgG titers (r = 0.56, p = 0.014) (Figure 6). A log_10_ increase in gp140 titers was associated with an absolute increase of 9.3 in % ADCC mediated killing by BMS (95% CI: 2.18, 16.41; p = 0.013).

### BMSADCC activity is associated with risk of infant transmission

The relationship between maternal clinical correlates and BMS Ab neutralization, HIV-env specific binding titers and ADCC activity were each individually assessed. There was no statistically significant association between antibody titers and any of the clinical parameters examined. (Table S3).

**Figure 7:**
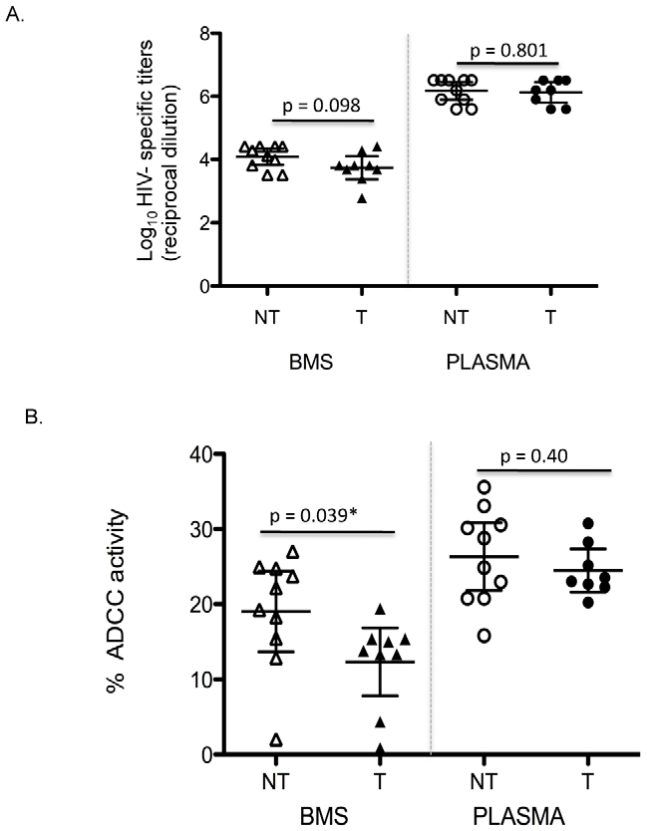
HIV-1 Env specific IgG titers and ADCC activity in BMS in relation to infant infection. (A) Relation of HIV-1 gp140 specific IgG titers and maternal transmission. Results are from duplicate testing of unfractionated BMS and plasma and are an average of at least two independent experiments. The Y-axis shows the log_10_ HIV-1 env specific IgG titers (reciprocal dilution) and the X-axis shows the sample type (BMS versus plasma) and HIV transmission status (NT versus T). (B) BMS and plasma mediated ADCC activity in relation to transmission. The Y-axis shows the % ADCC activity and the X-axis shows the sample type and HIV transmission status. In both panels, triangles and circles represent BMS and plasma, respectively while open and filled symbols represented non-transmitting and transmitting women, respectively. Results are from duplicate testing of unfractionated BMS and plasma and are an average of at least two independent experiments.

There was no statistically significant association between detection of NAbs and infant infection (OR = 0.31; 95% CI: 0.0050, 4.94; p = 0.58). We observed a trend for statistical significance between infant infection and reduced BMSgp140 HIV-1 env specific IgG titers but not plasma titers (estimated mean log_10_ difference 0.35 95% CI: −0.07, 0.77; p = 0.098) in a univariate analysis (Figure 7A). This association was in similar direction after controlling for plasma viral load(p = 0.038). Importantly, NT women were more likely to have higher BM ADCC activity compared to T women (estimated mean % killing difference 6.89; 95% CI: 0.41, 13.37; p = 0.039) (Figure 7B). This relationship remained significant in a multivariate analysis controlling for plasma viral load (p = 0.011) and both plasma and BM viral load (P = 0.012). There was no association between BM RNA viral load and BM ADCC activity (p = 0.520) in these 19 women. There was also no significant difference between plasma ADCC in T and NT women (Figure 7B).

## Discussion

The potential of HIV-1 specific Absin BM to inhibit HIV-1 or impact transmission risk has not been well defined. Despite the fact that the levels of both IgG and IgA were low in BM compared to plasma, we observed a trend for inverse correlation between the levels of HIV-1 specific IgG and risk of infant infection in the 19 women examined here. The effect of these antibodies did not appear to be through neutralization, as only 4 of 19 women had any detectable neutralizing IgG or IgA Abs and there was no correlation between detection of NAb and risk of infant infection. Rather, the important functional activity of these antibodies was linked to ADCC activity, as there was a statistically significant inverse correlation between the levels of ADCC activity and risk of infant infection. These data suggests that antibodies capable of mediating ADCC may be one factor that impacts the risk of BM HIV-1 transmission.

We found that BM HIV-1 env-specific IgG titers were significantly higher than those of IgA but significantly lower when compared to IgG from matched plasma samples. A reduced IgA response at mucosal sites in HIV-1 infection is contrary to what is observed with mucosal responses to other pathogens but consistent with previous reports of a low HIV-1 specific binding IgA response in favor of IgG at various mucosal sites [73]–[76]. In general, low mucosal BM IgA might reflect an ability of HIV-1 to impair local immune responses as a means of evading the humoral immune system at the mucosal site. However, the observation that BM HIV-1 env specific IgG titers were correlated with total plasma IgG levels suggests that some of the BM IgG may originate from systemic circulation, a process that could help fight infection at the mucosal site.

Despite low HIV-specific antibody levels in BMS compared to plasma, antibodies capable of ADCC were detected in all BMS samples. We found that the capacity to mediate ADCC was associated with the levels of HIV-1 env specific IgG titers, which is in agreement with data from previous studies [55], [77]–[79]. This is perhaps not surprising given that envelope binding is a required step for ADCC activity measured in the assay used here. Using purified BMS antibodies from a subset of these women, we further confirmed that ADCC activity in BM was exclusively mediated by IgG (data not shown). Thus, IgG mediated ADCC can be detected in unfractionated breastmilk, which includes IgA and other factors, as well as with purified antibody. ADCC titers have previously been shown to be generally higher compared to NAbs titers in the same individual possibly due to the specificity required to overcome the constraints posed by env protein in a bid to escape neutralization and also the fact that virus neutralization requires that all of the functional trimers be occupied by at least one antibody [80], [81]. Thus it may be possible to elicit high levels of antibodies capable of ADCC using an HIV-specific immunogen even in cases where neutralizing responses are limited.

BMS ADCC activity was significantly greater in NT compared to T women, suggesting a possible role in impacting infant infection. The mechanism by which BM ADCC might reduce transmission remains to be determined. ADCC would be expected to lead to effective clearance of infected cells. Given that the levels of HIV-infected cells in BM are correlated with transmission risk [52], it is plausible that HIV-specific ADCC responses within BM may act through reducing cell-associated viral transmission.

Other studies have implicated antibodies capable of ADCC in providing protection from infection and/or controlling an established infection. Several studies have shown that *de novo* ADCC responses to HIV and SIV infection are correlated with better viral control in chronic infection and/or clinical outcome. [77], [78], [82]–[85]. Vaccine-induced ADCC responses have also been correlated with reduced viral loads following SIV challenge [78], [79], [86]–[88], supporting a potential role of Fc-mediated antibody responses in blunting a new infection in SIV-infected macaques. A study by Forthal et al. also provided evidence that antibody-dependent cell-mediated virus inhibition, which is a measure of ADCC in combination with other antiviral activities, was correlated with infection rate in the Vax004 vaccine trial, although ADCC alone was not directly examined in this study [89]. In addition, studies of passive immunization using HIV monoclonal antibodies in macaques suggest that FcγR binding is required for optimal protective efficacy [90]. These findings support a potential role for antibodies that act through ADCC in providing protection from infection in the non-human primate model. The current study is the first that reports an association between HIV-specific ADCC activity and risk of HIV infection in humans.

This is the first study to examine BMS HIV-1 specific IgG and NAbs in relation to transmission risk using a relevant HIV-1 env representing recently transmitted virus from the dominant subtype in the population. This may explain our ability to detect a trend in association between binding antibodies and transmission, which was not seen in prior studies using other env proteins less representative of viruses in the study population to measure binding [47], [48].

We used the same highly neutralization sensitive (tier 1B) subtype A HIV-1 env representing the dominant subtype in the population under study to optimize our chances of detecting NAbs in BMS. Importantly, plasma from all subjects had a potent NAb response against this virus, indicating that all subjects had generated NAbs capable of specifically recognizing this test virus. Only 4 BMS had Abs that could neutralize >50% of either heterologous or autologous blood-derived viruses and the presence of HIV-1 specific NAbs was not associated with infant infection. The neutralizing activity was observed in women with higher levels of total IgG Abs in BMS. Therefore, it is possible that generally low IgG and IgA titers in BM might explain the limited neutralization capacity displayed by BM Abs.

The results of our study, showing low levels of HIV-1 env specific NAbs in BMS, are consistent with another recent study of BM HIV-1 NAbs [55]. In this study of a NVP-treated, clade C infected cohort, the levels of NAbs and HIV-1 env specific IgG were low in BM collected at 4 weeks post-delivery compared to plasma. We observed similarly low NAb levels in the breastmilk of ARV naïve women in a cohort that was enrolled prior to the availability of ARVs for prevention of MTCT [6]. Thus, collectively these studies indicate that the level of HIV-1 specific NAb are low in both early and mature milk, in both treated and untreated women and this is true no matter the infecting HIV-1 subtype.

We detected non-specific inhibition of HIV-1 and unrelated viruses (SIV, MLV) with several unfractionated BMS samples. This observation is perhaps not surprising because innate factors in BM such as defensins, lipids and lactofferin have documented activity against many viruses including enveloped retroviruses [4]. The ability of unfractionated BMS to inhibit HIV-1 in the in vitro TZM-bl assay used here did not correlate with risk of infant infection.

There are several limitations to our study, most notably the fact that we focused on a select group of women with high viral load and systemic NAbs in order to optimize our chances of detecting NAbs and to examine antibody levels in relation to transmission risk. Thus it is unknown if these findings are applicable to women with low viral loads or low systemic NAbs levels. Interestingly, a correlation between ADCC activity and viral control in SIV- infected macaques was only observed when animals with low viral load were excluded [86]. These authors suggested that a threshold of antigen may be needed to elicit robust ADCC. Certainly, larger studies using relevant env antigens to examine HIV-1 specific BM antibody responses in other populations will be needed to verify these findings and determine if the findings apply to women with lower viral levels and/or systemic NAb responses. In addition, while we focused on breastmilk antibodies in relation to post-partum transmission, there could be some misclassification of time of infection in this study. Specifically, the cases of transmission examined here were all cases of relatively early post-partum transmission and we cannot exclude that some were the result of intrapartum transmission, where BM antibody levels would be less relevant. Finally, while we did not see an association between BM viral RNA levels in this small study, but this does not rule out a relationship between ADCC and the cellular viral reservoir. Larger studies that include cell-associated virus levels and ADCC activity will be needed to clarify this issue.

In conclusion, we found that the capacity of BM to neutralize heterologous and autologous viruses obtained from blood and BM is limited. This observation can be explained in part by the low titers of Abs in BM compared to plasma in general, particularly IgG. It is unclear if such low NAb levels could play a role in protection, but no association was observed in this small study. However, the association between HIV-1 env specific IgG titers and ADCC activity with infant infection suggest that BM Ab could be playing some role in modulating infection through non-neutralizing mechanisms. To the best of our knowledge, this is the first study to report a positive association between BM transmission and ADCC capacity in BM. If these results are verified in a larger study of MTCT, then it would suggest that immunogens tailored at enhancing BM Abs capable of ADCC might be of potential benefit, particularly to HIV-1 infected women with high viral loads, who are at the greatest risk of transmission.

## Supporting Information

Figure S1 **Levels of HIV-1 env specific IgG (Y-axis) and IgA (X-axis) titers and detection of NAbs in BM.** Circles and squares represent transmitting and non-transmitting women, respectively. Symbols filled with black and grey correspond to detectable IgG and IgA neutralizing activity, while the open symbols denote no detection. One point might represent one or more values.
(TIF)

Table S1 **Neutralization potency of purified BMS IgG, IgA and FT.** The table shows the neutralization potency of purified BMS fractions from Transmitting (T) and Non-transmitting (NT) mothers against heterologous HIV and SIV as a negative control. Cases were assigned an IC50 value of <4 when neutralization was not detected.
(DOCX)

Table S2 **Summary of the relationships between total and HIV-1 env specific IgG and IgA in BMS and plasma.** Difference in levels (log_10_) indicates the average difference across the 19 women of the log_10_ levels for the first comparison measure minus the second. Correlations are described by the Pearson correlation coefficient, which indicates the strength of the linear association between the two variables, on the log_10_ scale. Plasma and BMS antibodies are correlated and lower levels were observed in BMS than in plasma. Env-specific IgG levels were greater than env-specific IgA.
(DOCX)

Table S3 **Summary of the associations for antibody levels and BM ADCC with clinical correlates of MTCT of HIV-1.** Estimates for each clinical correlate (maternal plasma viral load, breastmilk viral load and CD4 count) correspond to the estimated 10-fold change in the correlate with a unit increase in the row-level variable. Units for ADCC were on the absolute percentage scale, while units for all other variables were on the log_10_ scale. No statistically significant associations were observed.
(DOCX)
