# Supplementary figures and images for: HIV-Specific Antibodies Capable of ADCC Are Common in Breastmilk and Are Associated with Reduced Risk of Transmission in Women with High Viral Loads
Source: PLoS Pathog. 2012 Jun 14;8(6):e1002739. doi: 10.1371/journal.ppat.1002739 (PMC3375288; doi:10.1371/journal.ppat.1002739)

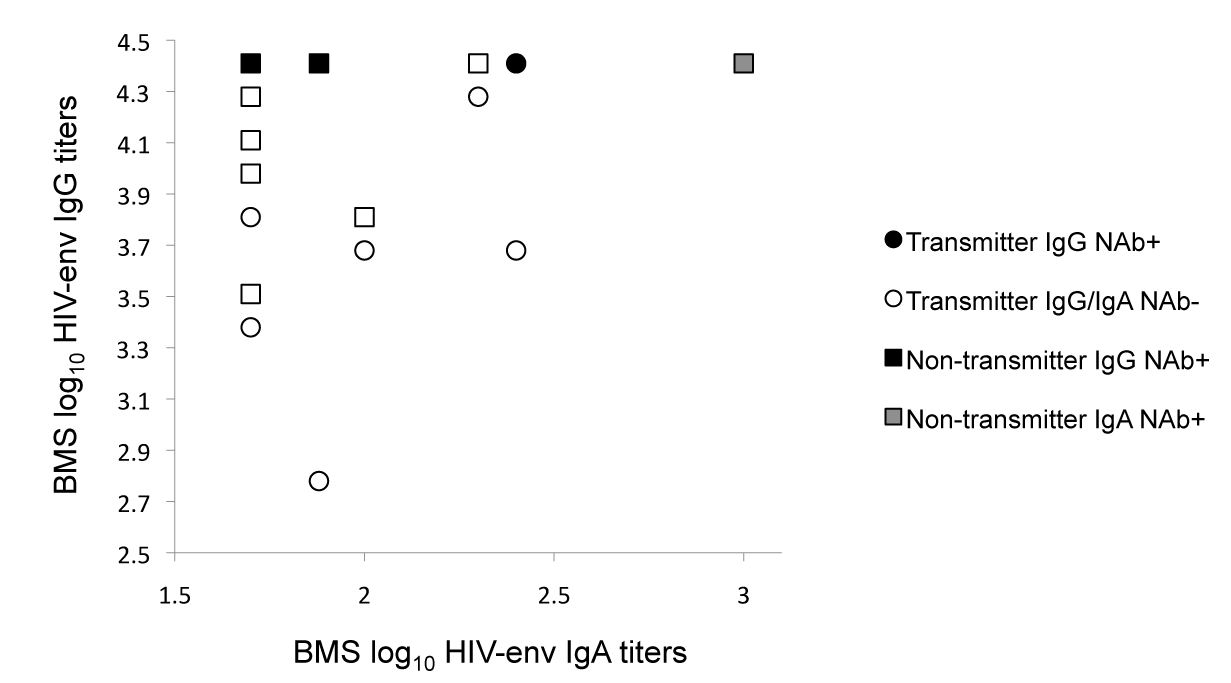

Supplement: Figure S1 — Levels of HIV-1 env specific IgG (Y-axis) and IgA (X-axis) titers and detection of NAbs in BM. Circles and squares represent transmitting and non-transmitting women, respectively. Symbols filled with black and grey correspond to detectable IgG and IgA neutralizing activity, while the open symbols denote no detection. One point might represent one or more values. (TIF) [file ppat.1002739.s001.tif]
