# Supplementary material for: HIV-Specific Antibodies Capable of ADCC Are Common in Breastmilk and Are Associated with Reduced Risk of Transmission in Women with High Viral Loads
Source: PLoS Pathog. 2012 Jun 14;8(6):e1002739. doi: 10.1371/journal.ppat.1002739 (PMC3375288; doi:10.1371/journal.ppat.1002739)
Supplement: Table S1 — Neutralization potency of purified BMS IgG, IgA and FT. The table shows the neutralization potency of purified BMS fractions from Transmitting (T) and Non-transmitting (NT) mothers against heterologous HIV and SIV as a negative control. Cases were assigned an IC50 value of <4 when neutralization was not detected. (DOCX) [file ppat.1002739.s002.docx]

|  |  | IgG IC50^a^ | | IgA IC50^a^ | | FT^b^ IC50^a^ | |
| --- | --- | --- | --- | --- | --- | --- | --- |
| ID Number | Visit Wk^c^ | HIV^d^ | SIV | HIV^d^ | SIV | HIV^d^ | SIV |
| Transmitting MB885 | 0 | <4 | <4 | <4 | <4 | **21** | **25** |
| Women MC046 | 0 | <4 | <4 | <4 | <4 | <4 | **10** |
| MF520 | 1 | <4 | <4 | <4 | <4 | <4 | <4 |
| MF535 | 14 | <4 | <4 | <4 | <4 | <4 | <4 |
| MI206 | 0 | <4 | <4 | <4 | <4 | **11** | **8** |
| MJ412 | 0 | <4 | <4 | <4 | <4 | **21** | **10** |
| MJ613 | 1 | <4 | <4 | <4 | <4 | <4 | <4 |
| MJ776 | 0 | **9.4** | <4 | <4 | <4 | <4 | <4 |
| MM596 | 6 | <4 | <4 | <4 | <4 | <4 | <4 |
| Non-Transmitting MA411 | 0 | <4 | <4 | <4 | <4 | **48** | **17** |
| Women MB727 | 8 | <4 | <4 | <4 | <4 | <4 | <4 |
| MB807 | 0 | <4 | <4 | <4 | <4 | <4 | <4 |
| MG540 | 0 | <4 | <4 | <4 | <4 | <4 | <4 |
| MH820 | 14 | <4 | <4 | <4 | <4 | **25** | **12** |
| MK371 | 2 | <4 | <4 | <4 | <4 | <4 | <4 |
| ML055 | 0 | <4 | <4 | <4 | <4 | <4 | <4 |
| ML267 | 0 | <4 | <4 | <4 | <4 | <4 | <4 |
| MM471 | 8 | <4 | <4 | <4 | <4 | <4 | <4 |
| MP199 | 6 | **9.9** | <4 | <4 | <4 | <4 | <4 |

^a^Purified antibody fractions neutralization assays were done at a starting dilution of 1:8; an IC50 of 4 was assigned in cases where 50% neutralization was not achieved.

^b^BMS flow through obtained after both IgG and IgA purification.

^c^Indicates time-point after delivery at which BM sample was obtained.

^d^Q461.d1
