# Supplementary material for: HIV-Specific Antibodies Capable of ADCC Are Common in Breastmilk and Are Associated with Reduced Risk of Transmission in Women with High Viral Loads
Source: PLoS Pathog. 2012 Jun 14;8(6):e1002739. doi: 10.1371/journal.ppat.1002739 (PMC3375288; doi:10.1371/journal.ppat.1002739)
Supplement: Table S2 — Summary of the relationships between total and HIV-1 env specific IgG and IgA in BMS and plasma. Difference in levels (log10) indicates the average difference across the 19 women of the log10 levels for the first comparison measure minus the second. Correlations are described by the Pearson correlation coefficient, which indicates the strength of the linear association between the two variables, on the log10 scale. Plasma and BMS antibodies are correlated and lower levels were observed in BMS than in plasma. Env-specific IgG levels were greater than env-specific IgA. (DOCX) [file ppat.1002739.s003.docx]

|  | Difference in levels (log_10_) | | Correlations | |
| --- | --- | --- | --- | --- |
|  | Estimate (95% CI) | P value | r (95% CI) | P value |
| BMS^a^ total IgG and IgA | -0.88 (-1.17, -0.60) | < 0.0001 | 0.39 (-0.09, 0.73) | 0.1049 |
| Plasma total IgG and IgA | 1.02 (0.88, 1.17) | < 0.0001 | 0.42 (-0.09, 0.76) | 0.1036 |
| Plasma and BMS total IgG | 2.25 (2.09, 2.40) | < 0.0001 | 0.67 (0.28, 0.87) | 0.0034 |
| Plasma and BMS total IgA | 0.39 (0.15, 0.62) | 0.0035 | 0.78 (0.47, 0.92) | 0.0003 |
| BMS env IgG and IgA | 1.96 (1.74, 2.19) | < 0.0001 | 0.38 (-0.10, 0.71) | 0.1133 |
| Plasma env IgG and IgA | 3.63 (3.38, 3.88) | < 0.0001 | 0.44 (-0.04, 0.75) | 0.0688 |
| Plasma and BMS env IgG | 2.22 (2.09, 2.35) | < 0.0001 | 0.81 (0.55, 0.93) | < 0.0001 |
| BMS env and total IgG | na^b^ | na | 0.76 (0.45, 0.90) | 0.0003 |
| Plasma and BMS env IgA | 0.59 (0.31, 0.87) | 0.00035 | 0.25 (-0.24, 0.64) | 0.3117 |
| BMS env and total IgA | na | na | 0.69 (0.33, 0.88) | 0.0015 |

^a^Breast milk supernatant

^b^Not Applicable
