# Supplementary material for: HIV-Specific Antibodies Capable of ADCC Are Common in Breastmilk and Are Associated with Reduced Risk of Transmission in Women with High Viral Loads
Source: PLoS Pathog. 2012 Jun 14;8(6):e1002739. doi: 10.1371/journal.ppat.1002739 (PMC3375288; doi:10.1371/journal.ppat.1002739)
Supplement: Table S3 — Summary of the associations for antibody levels and BM ADCC with clinical correlates of MTCT of HIV-1. Estimates for each clinical correlate (maternal plasma viral load, breastmilk viral load and CD4 count) correspond to the estimated 10-fold change in the correlate with a unit increase in the row-level variable. Units for ADCC were on the absolute percentage scale, while units for all other variables were on the log10 scale. No statistically significant associations were observed. (DOCX) [file ppat.1002739.s004.docx]

|  | Plasma Viral Load | | Breastmilk Viral Load | | CD4 Count | |
| --- | --- | --- | --- | --- | --- | --- |
|  | (log_10_) | | (log_10_) | | (log_10_) | |
|  | Estimate (95% CI^b^) | p value^c^ | Estimate (95% CI) | p value^c^ | Estimate (95% CI) | p value^c^ |
| Total BMS^a^ IgG | 0.19 (-0.30, 0.69) | 0.421 | 0.08 (-0.20, 0.36) | 0.566 | 0.27 (-0.59, 1.13) | 0.518 |
| Total Plasma IgG | 0.08 (-0.10, 0.26) | 0.355 | -0.05 (-0.16, 0.05) | 0.273 | 0.02 (-0.29, 0.34) | 0.871 |
| HIV BMS IgG | 0.36 (-0.24, 0.96) | 0.222 | 0.22 (-0.10, 0.55) | 0.169 | -0.05 (-1.13, 1.02) | 0.916 |
| HIV Plasma IgG | 0.13 (-0.40, 0.66) | 0.608 | 0.14 (-0.16, 0.43) | 0.342 | -0.43 (-1.32, 0.46) | 0.319 |
| Total BMS IgA | -0.04 (-0.91, 0.82) | 0.914 | 0.12 (-0.36, 0.60) | 0.615 | -1.08 (-2.45, 0.30) | 0.116 |
| Total Plasma IgA | 0.01 (-0.43, 0.44) | 0.979 | 0.04 (-0.21, 0.28) | 0.740 | -0.58 (-1.29, 0.13) | 0.103 |
| HIV BMS IgA | 0.24 (-0.26, 0.73) | 0.326 | 0.13 (-0.15, 0.40) | 0.335 | -0.47 (-1.32, 0.37) | 0.254 |
| HIV Plasma IgA | 0.31 (-0.42, 1.05) | 0.377 | 0.21 (-0.20, 0.62) | 0.295 | -0.30 (-1.59, 1.00) | 0.633 |
| BMS ADCC | 6.47 (-3.46, 16.39) | 0.187 | 1.78 (-3.93, 7.49) | 0.520 | 13.54 (-3.12, 30.19) | 0.105 |

^a^Breast milk supernatant

^b^Confidence Interval

^c^p values were by Welch’s unequal variance t-tests
